# Evaluation of the Role of Veterinarians for Outcomes Related to the Health and Production of Dairy Small Ruminants in Greece

**DOI:** 10.3390/ani13213371

**Published:** 2023-10-30

**Authors:** Daphne T. Lianou, George C. Fthenakis

**Affiliations:** Veterinary Faculty, University of Thessaly, 43100 Karditsa, Greece

**Keywords:** epg counts, farmer, mastitis, veterinarian, veterinary practice, veterinary profession, veterinary specialisation

## Abstract

**Simple Summary:**

This study, which was carried out as a large-scale investigation in 444 farms throughout Greece, explores the beneficial effects to small ruminant dairy farms of a steady and professional relationship with a veterinarian. The findings of detailed analyses indicated that, in summary, these benefits related to lower parasitic burden in animals of the farms and higher production of better-quality milk, as shown by the assessment of outcomes related to the health and production of animals. Moreover, the welfare of the animals in the farms was also improved, as evidenced by the lower incidence of painful diseases (e.g., clinical mastitis) and the effective use of relevant pharmaceutical products (e.g., non-steroid inflammatory drugs). The results attest that the application of veterinary advice and veterinary clinical services in sheep and goat dairy farms contributes to the improved health, production and welfare of animals.

**Abstract:**

The objective of the present study was to evaluate the potential benefits of veterinarians in improving the health and welfare of dairy sheep and goats by studying the associations of management practices employed in the farms with production- or health-related outcomes in sheep and goat farms in Greece. This work explored associations with ‘professional relationship with a veterinarian’ at 444 small ruminant dairy farms in an investigation performed around Greece, where 106 variables, related to infrastructure, animals, production outcomes, health management, health problems and human resources, were assessed. In 384 (86.5%) farms, a professional relationship with a veterinarian was maintained. The median value of visits made annually by veterinarians to these farms was five. In farms with a professional relationship with a veterinarian, significant differences were found in 24 variables (35.8%) related to management practices and 6 (30.0%) production- or health-related outcomes. In multivariable analysis, the following emerged with a significant association: epg counts in faecal samples (*p* = 0.014), average annual milk production per ewe/doe (*p* = 0.015), somatic cell counts in bulk-tank milk (*p* = 0.037), and annual incidence of clinical mastitis (*p* = 0.044). Moreover, associations of the characteristics of veterinarians emerged with somatic cell counts in bulk-tank milk: the gender (*p* < 0.0001) and the age (*p* = 0.004) of the veterinarians. The results attest that the application of veterinary advice and clinical services in sheep and goat dairy farms contributes to the improved health, production and welfare of animals.

## 1. Introduction

Veterinarians can play a role in improving the health and welfare of small ruminants through the control of diseases in the farms by means of investigation of problems occurring therein and the application of various (preventive or reactive) interventions. Within their professional capacity, veterinarians are important stakeholders for the welfare of animals, in all their professional roles. For example, in the role of government officials, they participate in the formulation of welfare standards for the transport and slaughter of animals and thus create and assess the standards for a safe food supply to people; they also participate in the creation of codes of conduct for veterinarians, farmers, etc., to address the care of farm animals. In the role of academics, they are responsible for teaching animal welfare, as well as improving animal health, to undergraduate students. In the role of practitioners, they are responsible for explaining to farmers, animal carers and their clients, the importance of animal welfare and to educate them about the necessity to improve animal health and welfare, which is reflected in the quality of the meat and milk produced and ultimately in the income of the farms.

A recent (September 2021) topical search in the Web of Science, using the terms ‘[improv* AND veterinary*] AND [sheep OR goat*] AND [welfare]’ (* is used as a truncation symbol), revealed 73 relevant articles.

Veterinary interventions in small ruminant farms contribute to improving health and welfare of the animals therein. Examples of such interventions include the management of obstetrical problems [1,2], the control of ectoparasitic infestations (psoroptic mange [3,4,5] and cutaneous myiosis [3,6]), the establishment of veterinarian-related practices as welfare indicators in farms [7,8], the monitoring of biosecurity practices in farms [9,10,11], the improvement in neonatal survival [2], the control of foot-related lameness [12,13], the administration of local anaesthesia as a means of pain management [14,15], the development of guidelines for handling sheep and goats [16], and the regularisation of welfare requirements in small ruminant farms [17].

Notably, 35 of these articles (47.9%) have been published during the last five years, i.e., since August 2018, thus indicating the increasing interest in the topic. However, most of these studies have been carried out in countries with meat-production sheep farming systems: 21 (28.8%) in the United Kingdom and another 22 (30.1%) in other countries. In contrast, only 16 (21.1%) papers have originated from Mediterranean countries and referred to dairy production systems, indicating the paucity of relevant studies for dairy small ruminant farms.

Veterinary work with small ruminants occupies around 21% of the overall time of veterinarians in Europe [18], with the lowest proportion in Russia (6%) and the highest in Iceland (55%). In fact, small ruminant work is the third most important area of focus of veterinarians in the continent, after small animal and cattle work [18]. Hence, there is interest in understanding the benefits that veterinarians may bring to farms with which they have a professional association.

The objective of the present study was to evaluate the potential benefits of veterinarians in improving the health and welfare of dairy sheep and goats by studying associations with management practices employed in the farms and with production- or health-related outcomes in sheep and goat farms in Greece.

## 2. Materials and Methods

### 2.1. Study Design—Collection of Samples and Information

**Figure 1:**
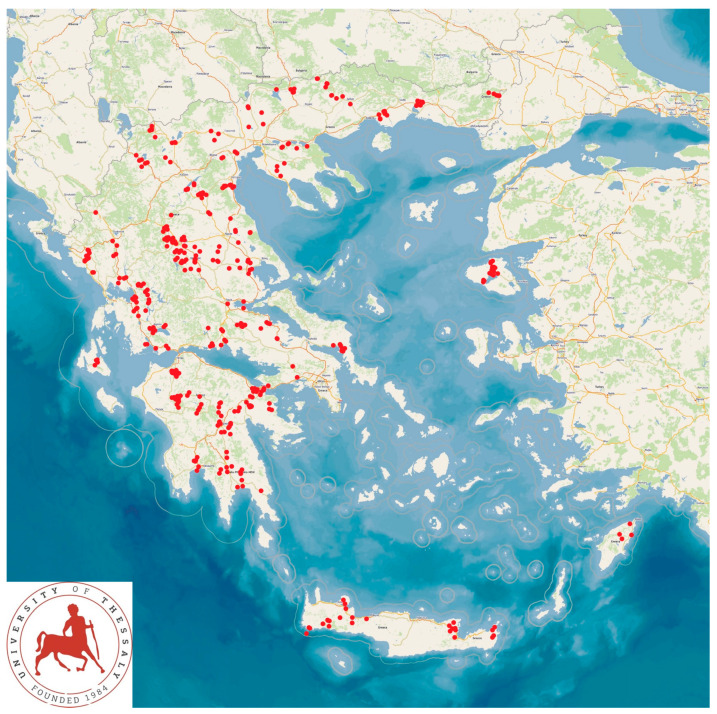
Locations (red dots) of 444 small ruminant dairy farms around Greece, in the 13 administrative regions of the country, which were visited during the countrywide investigation.

A large countrywide study was performed in 444 small ruminant farms in Greece (325 sheep flocks and 119 goat herds) (Figure 1). The farms were selected on a convenience basis, which referred to the eagerness and consent of the farmers to receive a visit for an interview and collection of samples. All farms were visited by the investigators in order to obtain information and samples. In total, these farms included 110,228 sheep and 30,192 goats [19].

Initially, an interview of the farmer was carried out, always by the same investigator (author D.T.L.). The senior investigator (author G.C.F.) introduced the interviewer to the farmer; he informed the farmers about her identity and her employment, as well as about the objectives of this study [19]. A detailed interview was carried out with the farmer, using a standardised, structured questionnaire, which included questions regarding infrastructure, animals, production outcomes, health management, health problems and human resources in the farm [19].

Then, on each farm visited, 25 female animals were randomly selected for body condition score evaluation. In order to adhere to the relevant standards [20] and to achieve uniformity of measurements, scoring (0–5, including half scores) was always performed by a certified European Veterinary Specialist in Small Ruminant Health Management.

Subsequently, samples from the bulk-tank milk of the farm were collected for further examinations. Bulk-tank milk samples were obtained for cytological, chemical and bacteriological examination by using aseptic methodology. The samples were collected using sterile plastic single-use pipettes, which were immersed into the tank to withdraw them [21].

Finally, faecal samples were collected directly from the rectum of adult female animals on the farms [21]. In each flock or herd, 20, 30, 40 or 50 ewes of female goats (for farms with ≤165, 166–330, 331–500 or >500 females, respectively) were selected for sampling.

### 2.2. Laboratory Examinations

Initially, bulk-tank milk samples were processed within 4 h of collection for somatic cell counting and measurement of milk composition (fat, protein, added water) by means of an automated counter, as detailed before [21].

Then, they were examined using microbiological techniques for total bacterial counting and for isolation of *Staphylococcus* spp. and *Listeria* spp. [21]. All staphylococcal isolates obtained were subjected to assessment for the detection of antibiotic resistance by employing the automated system BD Phoenix™ M50 [21].

Faecal samples were pooled and the McMaster technique was performed in quadruplicate samples obtained from these samples [21].

### 2.3. Data Management and Analysis

Data were entered into Microsoft Excel and analysed using SPSS v. 21 (IBM Analytics, Armonk, NY, USA). Initially, the farms were allocated into one of two cohorts: those that maintained a regular and professional relationship with veterinarians, or those that did not. In this context, ‘professional relationship’ referred to a stable, non-contractual, association with a veterinarian, who, in full accord with and by applying all the relevant professional veterinary conduct codes [22,23], was providing veterinary advice and clinical services in relation to the health and welfare of the animals in the farms.

Univariable analyses were performed initially. A ‘professional relationship with a veterinarian’ (as defined hereabove) at the farms was considered. A total of 106 variables were assessed (Table A1) and univariable analyses were performed. Comparisons between the results obtained for farms with or without a ‘professional relationship with a veterinarian’ were made by using the appropriate statistical methods, specifically, Pearson’s chi-squared test, Fisher exact test, z-test for proportions, analysis of variance or Mann–Whitney test, as appropriate. Then, parameters related to management practices and to production- or health-related outcomes that were found with a significance of *p* < 0.10 in the above analysis were further evaluated within the cohort of farms with a ‘professional relationship with a veterinarian’ for potential associations with the gender and the age of the veterinarians, as well as with the annual frequency of veterinary visits to the farm. For the evaluations for potential associations with the gender of the veterinarians, the same techniques as above were used as appropriate. For the evaluations for potential associations with the age of the veterinarians and the frequency of veterinary visits, Spearman’s rank correlation was employed.

The above were then followed by multivariable analyses. For the identification of potential associations of a ‘professional relationship with a veterinarian’ with production- or health-related outcomes, a multivariable model was constructed; variables (production- or health-related outcomes) found with *p* < 0.2 in the preceding univariable analysis were included into this model. Progressively, variables included into the multivariable model were removed from the model by using backwards elimination. The likelihood ratio test was performed to assess the *p*-value of each parameter; among those found with *p* ≥ 0.2, the one with the largest *p* was removed from the model. The procedure was repeated, until no variable with *p* ≥ 0.2 could be removed from the model [24]. The variables included in the final multivariable model constructed are in Table S1. After identifying in the multivariable analysis the production- or health-related outcomes that were significantly associated with the ‘professional relationship with a veterinarian’, a further multivariable analysis was performed to study associations with the gender and the age of the veterinarians, as well as with the annual frequency of veterinary visits to a farm for each of these outcomes. The variables included in the final multivariable models constructed are in Table S1.

For the results of somatic cell counts and total bacterial counts in milk, appropriate transformations to normalise the data were performed before the analysis [25,26]. For the evaluation of epg counts, only results from farms in which anthelmintic administration had not been performed during the two months prior to sampling (*n* = 369) were taken into account.

In all analyses, statistical significance was defined at *p* < 0.05.

## 3. Results

### 3.1. Descriptive Results

Of the 444 farms visited, in 384 (86.5%, 95% confidence interval (CI): 83.0–89.4%), the farmers indicated that they maintained a professional relationship with a veterinarian. The median value of visits made annually by veterinarians to these farms was 5 (interquartile range: 7).

Veterinary visits were also made to farms where farmers indicated that they did not maintain a professional relationship with a veterinarian. Nevertheless, their frequency was significantly lower; the median value of visits made annually by veterinarians to such farms was 2 (0.25) (*p* < 0.0001).

### 3.2. Characteristics of Veterinarians Associated with the Farms Visited

In total, the farmers maintained a professional relationship with 47 different veterinarians, 17 (36.2%) females and 30 (63.8%) males. The average age of these veterinarians was 42.8 ± 1.5 years.

Male veterinarians were significantly older than females: mean age was 45.4 ± 1.9 years versus 38.1 ± 2.1 years, respectively (*p* = 0.019). Among veterinarians younger than 35 years, there were more females (58.3%), whilst among veterinarians older than 50 years, there were more males (85.7%).

There was no difference between female and male veterinarians in the number of visits made to the farms annually: 5 (7) versus 5 (6.5), respectively (*p* = 0.82).

Most of the veterinarians (*n* = 29, 61.7%) were graduates of the Faculty of Veterinary Medicine of the Aristotle University of Thessaloniki, and fewer (*n* = 13, 28.7%) were graduates of the Veterinary Faculty of the University of Thessaly, whilst a smaller number were graduates of veterinary Faculties of other European countries (*n* = 5, 10.6%). Notably, 17 of the veterinarians (36.2%) had followed some postgraduate training in farm animal health management and diseases.

### 3.3. Differences between Farms with or without a Professional Relationship with a Veterinarian

The detailed results of the univariable analysis for the 106 variables are shown in Table S2. A significant difference between farms with or without a professional association with a veterinarian was found for 38 variables (Table A2).

With regard to variables related to management practices, there was a significant difference in farms with a professional relationship with a veterinarian for 24 (35.8%) practices; further, there was a tendency for significance for 8 (11.9%) practices (Table 1).

With regard to production- or health-related outcomes, there was a significant difference in farms with a professional relationship with a veterinarian for six (30.0%) outcomes, and there was a tendency for significance for one (5.0%) outcome (Table 1 and Table 2).

**Figure 2:**
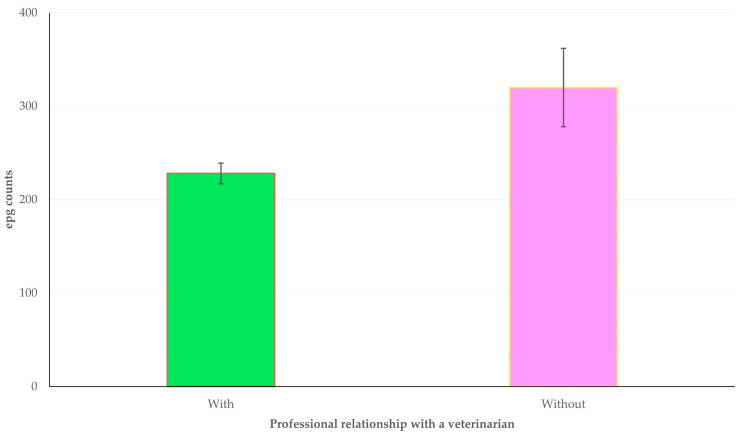
Mean epg counts in faecal samples among farms with (green bar) or without (pink bar) professional relationship with a veterinarian, as found in a countrywide cross-sectional study in 444 small ruminant farms in Greece.

**Figure 3:**
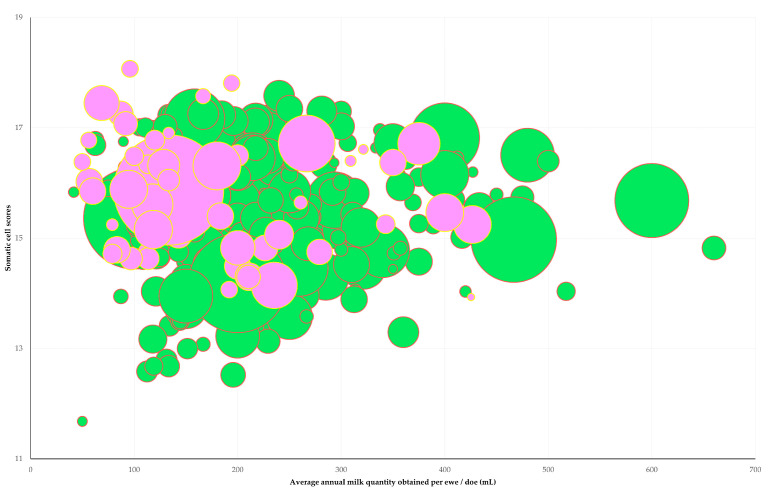
Average annual milk production per ewe/doe (horizontal axis), somatic cell counts in bulk-tank milk (vertical axis) and annual incidence of clinical mastitis (diameter of bubbles) among farms with (green-coloured bubbles) or without (pink-coloured bubbles) a professional relationship with a veterinarian, as found in a countrywide cross-sectional study in 444 small ruminant farms in Greece.

In the multivariable analysis, the following production- or health-related outcomes emerged with a significant association with a professional relationship with a veterinarian (Table 3): (a) epg counts in faecal samples (*p* = 0.012) (Figure 2); (b) average annual milk production per ewe/doe (*p* = 0.015); (c) somatic cell counts in bulk-tank milk (*p* = 0.037); and (d) annual incidence of clinical mastitis (*p* = 0.044) (Figure 3).

### 3.4. Differences among Farms with a Professional Relationship with a Veterinarian, in Accord with Characteristics of the Veterinarian

#### 3.4.1. Gender of the Veterinarian

The detailed results of the univariable analysis for association of management practices and production- or health-related outcomes with the gender of the veterinarian are in Table S3. A significant difference between farms related to the gender of the veterinarian with whom there was a professional association was found for ten management practices (*p* ≤ 0.028 for all relevant comparisons; details are in Table S3) and for two production- or health-related outcomes (*p* ≤ 0.026 for all relevant comparisons; details are in Table 4).

#### 3.4.2. Age of the Veterinarian

The detailed results of the univariable analysis for the association of management practices and production- or health-related outcomes with the age of the veterinarian are in Table S4. A significant correlation in accord with the gender of the veterinarian, with whom there was a professional association, was found for six variables related to management practices (*p* ≤ 0.029 for all relevant comparisons; details are in Table S4) and for two production- or health-related outcomes (*p* ≤ 0.024 for all relevant comparisons; details are in Table 5).

#### 3.4.3. Annual Frequency of Veterinary Visits to the Farms

The detailed results of the univariable analysis for the association of management practices and production- or health-related outcomes with the annual frequency of veterinary visits to the farms are in Table S5. A significant correlation in accord with the annual frequency of veterinary visits to the farms was found for 16 variables related to management practices (*p* ≤ 0.045 for all relevant comparisons; details are in Table S5); however, a significant correlation was not seen for any production- or health-related outcome (*p* > 0.07 for all relevant comparisons; details are in Table S5).

### 3.5. Associations of Characteristics of Veterinarians with Production- or Health-Related Outcomes

**Figure 4:**
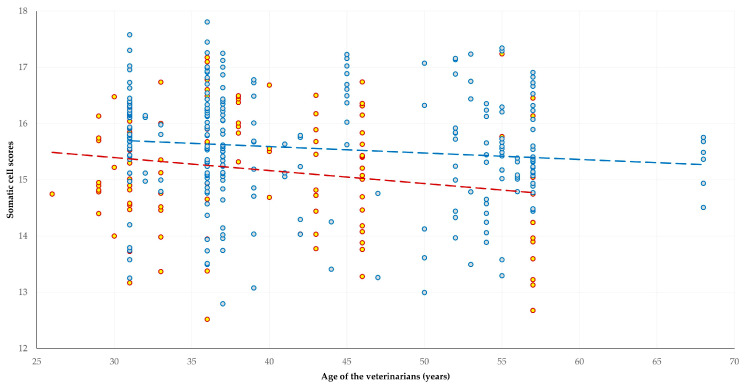
Somatic cell counts in the bulk-tank milk, in accord with the gender (red-yellow dots: female; blue-grey dots: male) and the age of the veterinarians among farms with a professional relationship with a veterinarian, as found in this countrywide cross-sectional study among 384 small ruminant farms in Greece (red and blue dashed lines are respective trendlines).

In the multivariable analyses performed, significant associations of the characteristics of veterinarians emerged only for the somatic cell counts in bulk-tank milk, specifically, related to a) the gender (*p* = 0.0001) and b) the age (*p* = 0.007) of the veterinarians (Table 6, Figure 4).

For the other production- or health-related outcomes, no significant associations with the characteristics of veterinarians emerged, i.e., for the epg counts in faecal samples (*p* > 0.06), the annual incidence of clinical mastitis (*p* > 0.11), or the average annual milk production per ewe/doe (*p* > 0.13).

## 4. Discussion

### 4.1. Associations of Professional Relationship with a Veterinarian with Outcomes Related to Milk Production

The findings indicate that the beneficial effects were focused on the production of milk in the farm, as milk production, somatic cell counts in bulk-tank milk and incidence of clinical mastitis were three outcomes significantly improved in farms with a professional relationship with a veterinarian. The improved outcomes regarding milk quantity and quality are the result of the application of a variety of targeted management practices by the veterinarians: preventive use of laboratory diagnostic examinations in samples of milk, vaccination against contagious agalactia, vaccination against staphylococcal mastitis, administration of flunixin in cases of clinical mastitis, and improved general management practices (e.g., better nutritional management). These findings are in line with the production system prevalent in the country, i.e., dairy production [27], as well as with the farmers’ consideration of mastitis as the most important problem in their flocks/herds [21]. Therefore, veterinarians comply with the requirements of their clients and contribute to the increase in agricultural production (animal production) relevant to the country.

It is also noteworthy that in a recent scientometrics study of mastitis in sheep [28], the two veterinary faculties of Greece were among the top three establishments internationally with regard to research output on that subject, whilst in another evaluation, it was found that research about sheep and goats in Greece has focused on milk production and diseases of the udder of small ruminants [29]. This indicates the increased interest in the study and control of the infection in the country, as well as the production of relevant knowledge, which is disseminated to field practicing veterinarians. These, in turn, usefully apply that knowledge to the field.

### 4.2. Associations of Professional Relationship with a Veterinarian with Practices Related to Administration of Pharmaceutical Products

In Greece, veterinarians active in small ruminant health management make most of their income through the sale of veterinary pharmaceuticals, for which they have the exclusive right. Indeed, veterinary services to small ruminant farmers are mostly provided for ‘free’, considered as a ‘professional gift’ for the purchase of veterinary products.

The above is reflected in the findings of the evaluation of parameters related to the administration of pharmaceutical products: higher number of occasions of administration of anthelmintic drugs annually, more frequent prescription of (more expensive) injectable solutions for anthelmintic use, more common routine administration of antimicrobials to newborns, more frequent administration of selenium to newborn animals, and more frequent use of flunixin in the treatment of clinical mastitis. Whilst, in some cases, there can be a benefit for these, the financial aspect might always be involved in the decision for prescribing the respective drugs.

The increased prescription and administration of antibiotics to newborns can lead to the development of antibiotic resistance [30,31,32,33] and it must thus be discouraged. Moreover, the present results did not show that the more frequent administration of antibiotics to newborns was associated with a lower incidence of pneumonia on diarrhoea in newborns, which are the most significant problems of lambs/kids at that age [34,35,36,37]. It is also noted that in sheep and goat farms in Greece, determinants of the administration of antibiotics in the treatment of various infections were found to mostly be the socio-demographic characteristics of farmers rather than management- or animal-related factors in the farms [38]. An improved use of antimicrobials, as underlined by scientific principles and compliance with policies and regulations, is important for an improvement in the welfare of the sheep and goats, as well as for reducing the risk for the development of antibiotic resistance.

The increased number of occasions of anthelmintic administration would have contributed to the lower epg counts found in these farms. Nevertheless, the frequent administration of anthelmintics is a main risk factor for the development of resistance by gastrointestinal nematodes [39,40,41] and this might have contributed to the presence of extensive and countrywide resistance of *Haemonchus contortus* to benzimidazoles, as found in a recent relevant field investigation [42]. One can also postulate that the frequent prescription of macrocyclic lactones by veterinarians might be practiced as a consequence of the understanding of veterinarians of the possibility of the existence of that widespread resistance.

There are nevertheless some positive facets in this increased administration of pharmaceutics. It is noted that among farms with a professional association with a veterinarian, more frequent use of flunixin was also made. Flunixin contributes to a reduction in the clinical signs of mastitis and alleviates pain [43,44], thus improving the welfare of the animals. That way, veterinarians also contribute to improving animal welfare, given that mastitis has been determined by the European Food Safety Authority to be a disease significantly reducing sheep welfare [45].

All of the above confirm the need to continue the training of professionals and of farmers in the correct usage of veterinary pharmaceuticals. Correct usage should be guided by scientific knowledge and surveying works, with the aim to contribute to a reduction in the resistance of the various pathogens (antibiotics, anthelmintics).

### 4.3. Characteristics of the Veterinarians

The majority of the veterinarians involved in this study were male. However, the female veterinarians were younger than the male ones, which is a consequence, in Greece and internationally, of most veterinary students over the last 20 years being females. In some faculties, this proportion can even be up to 80% of the total students [18,46,47].

Whilst farm animal veterinary work had been previously considered to be a male-dominated focus of the profession, this has evidently been changing, despite the fact that female veterinary students do not frequently consider such a career [48]. Possibly, this may be due to the changing landscape of veterinary work with farm animals, which nowadays involves an increased advisory and preventive farm health approach rather than clinical work at the individual animal level [48,49]. Additionally, it is also noted that, Europe-wide, of the 54 listed European Veterinary Specialists in Small Ruminant Health Management, 50% are females [50].

In farms with a professional relationship with a female veterinarian, some health-related outcomes were better than in farms with a relationship with a male veterinarian. The final grades of the veterinary degree can be considered a reflection of the knowledge acquired by young graduates during their studies and may thus represent the cognitive level of a new graduate regarding veterinary work [51,52]; this may affect professional actions, including health management in farms. It is thus interesting that an analysis of the final grades of graduates of the Veterinary Faculty of the University of Thessaly revealed that, during the period of 1999 to 2023, female veterinarians graduated with an overall higher final grade than male veterinarians: 6.65 ± 0.02 versus 6.46 ± 0.03 (average ± standard error of the mean; maximum possible: 10), respectively (*p* < 0.0001).

With regard to age, the application of more frequent management practices by younger veterinarians can be in line with more recent relevant scientific developments. For example, the benefits of the administration of selenium to pregnant ewes/does have only been described in Greece in the last few years [53,54] and have now been disseminated to practicing veterinarians. The present findings are in line with a report that older sheep farmers use fewer of the various management tools available to improve the health of the animals in their farms and less frequently [55].

## 5. Conclusions

Veterinary practitioners active in farm animal practice have a significant responsibility to the farmers and to the animals within these farms. Their work involves the maintenance of animal health and animal welfare in the farms, and, within this frame, they discuss with farmers the appropriate management practices to improve the health of the animals, to mitigate disease and distress (including pain control) and to maximise productivity.

The results attest that the application of veterinary advice and clinical services in sheep and goat dairy farms contributes to the improved health, production and welfare of the animals. Nevertheless, the training of veterinary practitioners in the correct use of pharmaceuticals should continue, especially given the European initiatives to minimise the administration of drugs in farm animals [56].

Whilst the results provide clear evidence regarding the beneficial role of veterinarians in small ruminant farms, it should be noted that veterinary services to these farmers are underpaid or even not paid at all. Farmers view the veterinary work in their flocks/herds as a ‘perk’ or ‘free benefit’ carried out by veterinarians in association with the drugs (pharmaceutical and immunological products) that they sell to the farmers. This may lead to the minimisation of the services provided, especially as veterinary drugs necessary for the farms can be sold to farmers by various veterinarians, even ones that have not provided clinical services to the animals of a farm.

This study presented another facet of the interactions between people and farm animals within the food-producing chain. These people–animal interactions can possibly be considered another approach within the ‘One Health’ concept.

## Figures and Tables

**Table 1:** Summary of the significance in variables related to management practices and production- or health-related outcomes found in 444 small ruminant farms in Greece, in accord with professional relationship with a veterinarian.

Type of Variables	Difference between Farms with or without Professional Relationship with a Veterinarian in Number of Variables		
	Significant ^1^	Tending for Significance ^2^	Not Significant ^3^
Health management	8	2	9
Pharmaceutical treatment	3	0	4
Biosecurity management	2	2	4
Reproductive management	5	3	10
Management in the milking parlour	1	0	5
Nutritional management	5	1	3
Production-related outcomes	2	0	3
Health-related outcomes	3	1	10
Animal condition outcomes	1	0	0
Total	30 variables	9 variables	48 variables

^1^: *p* < 0.05; ^2^: 0.05 ≤ *p* < 0.10; ^3^: *p* ≥ 0.10.

**Table 2:** Production- or health-related outcomes found with a significant association with professional relationship with a veterinarian in this countrywide cross-sectional study in 444 small ruminant farms in Greece.

Farms in Which There Was a Professional Relationship with a Veterinarian (*n* = 384)	Farms in Which There Was No Professional Relationship with a Veterinarian (*n* = 60)	*p*-Value
Average milk production per ewe/doe during the preceding milking period		
199.5 (127.5) L	129.0 (114.0) L	<0.0001
Average number of lambs/kids born per ewe/doe		
1.28 (0.20) newborns	1.20 (0.16) newborns	0.003
Incidence of clinical mastitis during the preceding season		
2.0% (4.5%)	3.0% (4.1%)	0.021
Epg counts in faecal samples		
228 ± 11 epg	320 ± 42 epg	0.011
Body condition score		
2.40 (0.22)	2.26 (0.61)	0.016
Somatic cell counts in bulk-tank milk		
0.543 × 10^6^ (0.504 × 10^6^–0.583 × 10^6^) cells mL^−1^	0.680 × 10^6^ (0.567 × 10^6^–0.814 × 10^6^) cells mL^−1^	0.026

**Table 3:** Results of multivariable analysis of the professional relationship with a veterinarian with production- or health-related outcomes in this countrywide cross-sectional study in 444 small ruminant farms in Greece.

Production- or Health-Related Outcomes	Odds Risk (±se ^1^)	*p*
epg counts in faecal samples		0.012
With ‘veterinarian’ ^2^	reference	-
Without ‘veterinarian’	1.406 ± 0.147	0.011
Average annual milk production per ewe/doe		0.015
With ‘veterinarian’	reference	-
Without ‘veterinarian’	1.269 ± 0.109	0.002
Somatic cell counts in bulk-tank milk		0.037
With ‘veterinarian’	reference	-
Without ‘veterinarian’	1.021 ± 0.012	0.028
Annual incidence of clinical mastitis		0.044
With ‘veterinarian’	reference	-
Without ‘veterinarian’	1.408 ± 0.429	0.24

^1^: se: standard error; ^2^: i.e., a professional relationship with a veterinarian.

**Table 4:** Production- or health-related outcomes found with a significant association with the gender of the veterinarian with whom there was a professional relationship in a cross-sectional study in a countrywide cross-sectional study among 384 small ruminant farms in Greece.

Farms in Which There Was a Professional Relationship with a Female Veterinarian (*n* = 121)	Farms in Which There Was a Professional Relationship with a Male Veterinarian (*n* = 263)	*p*-Value
Epg counts in faecal samples		
183 ± 15 epg	250 ± 16 epg	0.024
Somatic cell counts in bulk-tank milk		
0.461 × 10^6^ (0.401 × 10^6^–0.529 × 10^6^) cells mL^−1^	0.585 × 10^6^ (0.540 × 10^6^–0.638 × 10^6^) cells mL^−1^	0.026

**Table 5:** Production- or health-related outcomes found with a significant association with the age of the veterinarian, with whom there was a professional relationship in a cross-sectional study in a countrywide cross-sectional study among 384 small ruminant farms in Greece.

Variables	*r_sp_*	Type of Association	*p*-Value
Incidence of clinical mastitis	0.116	positive	0.024
Epg counts in faecal samples	0.131	positive	0.020

**Table 6:** Results of multivariable analysis for variables related to the characteristics of veterinarians with a significant association with somatic cell counts in bulk-tank milk among 384 small ruminant farms in Greece.

Variables	Odds Risk (±se ^1^)	*p*
Gender of veterinarian		0.0001
Female	reference	-
Male	1.558 ± 1.120	-
Age of veterinarian		0.004
Per year decrease	1.015 ± 1.005	-

^1^: se: standard error of the mean.

## Data Availability

Data associated with this study are presented in the text or in the Supplementary Materials.

## Supplementary Materials

The following supporting information can be downloaded at: https://www.mdpi.com/article/10.3390/ani13213371/s1, Table S1: Details of multivariable models employed for the evaluation of professional relationship with production- or health-related outcomes in 325 sheep flocks and 119 goat herds in Greece; Table S2: Results of univariable analysis of parameters evaluated for association, in 325 sheep flocks and 119 goat herds in Greece, with the outcome ‘professional relationship with a veterinarian’; Table S3: Results of univariable analysis of parameters related to management practices and to production- or health-related outcomes evaluated for association, in 283 sheep flocks and 101 goat herds in Greece, with a ‘professional relationship with a veterinarian’, in accord with the gender of the veterinarian; Table S4: Results of univariable analysis of parameters related to management practices and to production- or health-related outcomes evaluated for association, in 283 sheep flocks and 101 goat herds in Greece, with a ‘professional relationship with a veterinarian’, in accord with the age of the veterinarian; Table S5: Results of univariable analysis of parameters evaluated for association, in 283 sheep flocks and 101 goat herds in Greece, with a ‘professional relationship with a veterinarian’, in accord with the annual frequency of veterinary visits to the farm.

## Appendix A

**Table A1:** Variables (*n* = 106) evaluated for potential association with professional relationship with a veterinarian in a cross-sectional study in 444 small ruminant farms in Greece.

**General Management Applied in the Farm**
Management system applied in farms (description according to EFSA classification: intensive/semi-intensive/semi-extensive/extensive)
Seasonal transfer of animals to other sites (yes/no)
**Infrastructure**
Availability of milking parlour (yes/no)
Availability of isolation facilities for animals (yes/no)
Availability of milk replacer facilities and equipment for administration of milk replacer (yes/no)
**Animals**
No. of female animals in the farm (no.)
Average age of culling females (years: up to 6 years of age/over 6 years of age)
**Health Management**
Preventive use of laboratory diagnostic examinations in samples of milk (yes/no)
Preventive use of laboratory diagnostic examinations in samples of blood (yes/no)
Preventive use of laboratory diagnostic examinations in samples of faeces (yes/no)
Number of occasions of administration of anthelmintic drugs annually (no.)
Families of anthelmintics administered (description)
Pharmaceutical form of anthelmintics administered (description)
Administration of ectoparasiticides (yes/no)
Vaccination against *Chlamydia* infection (yes/no)
Vaccination against clostridial infections (yes/no)
Vaccination against contagious agalactia (yes/no)
Vaccination against foot rot (yes/no)
Vaccination against contagious ecthyma (yes/no)
Vaccination against paratuberculosis (yes/no)
Vaccination against bacterial pneumonia (yes/no)
Vaccination against staphylococcal mastitis (yes/no)
Total number of optional vaccines administered annually (no.)
Administration of ‘dry-ewe’ treatment at the end of the lactation period (yes/no)
Duration of the dry period (months)
Record keeping (yes/no)
**Pharmaceutical Treatment**
Routine administration of antimicrobials to newborns (yes/no)
Maintenance of prescribed withdrawal periods after administration of pharmaceuticals (yes/no)
Means of calculating live bodyweight for the administration of pharmaceutical products (weighing/estimation)
Routine overdosing (compared to dose prescribed) of pharmaceuticals (yes/no)
Number of antibiotics used for treatment of clinical mastitis (number)
Route for administration of antibiotics (systemic/intramammary)
Administration of flunixin in cases of clinical mastitis (yes/no)
**Biosecurity Management**
Quarantine of new animals arriving at the farm (yes/no)
Isolation of sick animals at the farm (yes/no)
Means for disposal of carcasses of animals that died in the farm (incineration, burial or removal by specialised agent/given to dogs, left unburied, or left in water streams)
Presence of a ditch at the entrance of the farm (yes/no)
Presence of a fence or a wall around the farm (yes/no)
Carrying out disinfections in the farm (yes/no)
Practicing sharing of equipment with other farms (yes/no)
Administration of rodenticides (yes/no)
Presence of spots suitable for reproduction of vectors within 500 m (yes/no)
**Reproductive Management**
Beginning of the mating period for ewes/does (month)
Application of reproductive control (yes/no)
Changes of rams/bucks into ewes/does during the mating period (yes/no)
Use of artificial insemination (yes/no)
Use of embryo transfer (yes/no)
Use of ultrasound for pregnancy diagnosis (yes/no)
Nutritional modifications before the lambing/kidding period (yes/no)
Grouping of pregnant females during the final stage of pregnancy (yes/no)
Administration of oxytetracycline to the pregnant animals (yes/no)
Administration of selenium to pregnant animals (yes/no)
Induction of lambing/kidding (yes/no)
Recording of births—maintenance of a lambing/kidding book (yes/no)
Newborn care and specific monitoring (yes/no)
Month of the start of the lambing/kidding season (description)
Administration of selenium to newborn animals (yes/no)
Disinfection of navel stump in newborns (yes/no)
Maintenance of a colostrum bank (yes/no)
Tail docking in newborns (yes/no)
Newborn fostering to female animals other than their dams (yes/no)
Age of lamb/kid removal from their dams (days)
**Management in the Milking Parlour**
Daily number of milking sessions (no.)
System pulsation rate (p. min^−1^)
System pressure (kPa.)
Use of teat disinfection after milking (yes/no)
Temperature of cleaning water after the milking sessions (°C)
Frequency of changing teatcups (description)
**Nutritional Management**
Provision of hay as fodder to animals (yes/no)
Average quantity of hay provided daily to animals during the preceding season (kg)
Provision of straw to animals (yes/no)
Provision of silage to adult animals (yes/no)
Provision of finished feed (concentrate) to adult animals (yes/no)
Provision of finished feed (concentrate) to adult animals throughout the year (yes/no)
Type of finished feed (concentrate) provided to adult animals (description)
Average quantity of finished feed (concentrate) provided daily to animals during the preceding season (kg)
Person responsible for nutritional management (description)
**Production- or Health-related Outcomes**
Average milk production per ewe/doe during the preceding milking period (litres)
Average number of lambs/kids born per ewe/doe (no.)
Incidence of clinical mastitis during the preceding season (%)
Incidence of abortion during the preceding season (%)
Incidence of lameness during the preceding season (%)
Incidence of mange during the preceding season (%)
Incidence of obstetrical problems during the preceding season (%)
Incidence of deaths, of any cause, in adult animals during the preceding season (%)
Incidence of pneumonia in lambs/kids during the preceding season (%)
Incidence of diarrhoea in lambs/kids during the preceding season (%)
Epg counts in faecal samples (epg)
Body condition score (score on scale 0–5, including half-scores)
Somatic cell counts in bulk-tank milk (cells mL^−1^)
Total bacterial counts in bulk-tank milk (cfu mL^−1^)
Isolation of staphylococci from bulk-tank milk (yes/no)
Isolation of antibiotic-resistant staphylococci from bulk-tank milk (yes/no)
Isolation of *Listeria* spp. from bulk-tank milk (yes/no)
Fat content in bulk-tank milk (%)
Protein content in bulk-tank milk (%)
Added water in bulk-tank milk (%)
**Characteristics of Human Resources**
Age of farmer (years)
Length of previous animal farming experience (years)
Farmer’s general education (description: primary = European Qualifications Framework Levels 1 or 2/secondary or post-secondary = European Qualifications Framework Levels 3, 4 or 5/tertiary = European Qualifications Framework Level 6, 7 or 8)
Farmer’s professional involvement in farming (full-time/part-time)
Daily period spent by farmer at the farm (hours)
Family tradition in farming (yes/no)
Presence of working staff in the farm (yes/no)
Occurrence of brucellosis in farmer (yes/no)

## Appendix B

**Table A2:** Variables (*n* = 37) found with a significant association with professional relationship with a veterinarian in a cross-sectional study in 444 small ruminant farms in Greece.

Farms in Which There Was a Professional Relationship with a Veterinarian (*n* = 384)	Farms in Which There Was No Professional Relationship with a Veterinarian (*n* = 60)	*p*-Value
Management system applied in farms		
Intensive: 12.5%, Semi-intensive: 40.4%, Semi-extensive: 40.9%, Extensive: 6.3%	Intensive: 8.3%, Semi-intensive: 23.3%, Semi-extensive: 33.3%, Extensive: 35.0%	<0.0001
Seasonal transfer of animals to other sites		
Yes: 15.9%, No: 84.1%	Yes: 28.3%, No: 81.7%	0.018
Availability of milking parlour		
Yes: 76.0%, No: 24.0%	Yes: 48.3%, No: 51.7%	<0.0001
Availability of isolation facilities for animals		
Yes: 77.1%, No: 22.9%	Yes: 55.0%, No: 45.0%	0.0003
Average age of culling females		
≤6 years: 62.2%, >6 years: 37.8%	≤6 years: 50.0%, >6 years: 50.0%	0.007
Preventive use of laboratory diagnostic examinations in samples of milk		
Yes: 23.7%, No: 76.3%	Yes: 6.7%, No: 93.3%	0.0003
Preventive use of laboratory diagnostic examinations in samples of faeces		
Yes: 13.3%, No: 86.7%	Yes: 0.0%, No: 100.0%	0.003
Number of occasions of administration of anthelmintic drugs annually		
2 (1)	1.5 (1)	0.009
Pharmaceutical form of anthelmintics administered		
Injectable solution: 60.7%	Injectable solution: 38.3%	0.001
Vaccination against contagious agalactia		
Yes: 61.2%, No: 38.8%	Yes: 26.7%, No: 73.3%	<0.0001
Vaccination against staphylococcal mastitis		
Yes: 39.1%, No: 60.9%	Yes: 16.7%, No: 83.3%	0.0007
Total number of optional vaccines administered annually		
3 (2)	2 (2)	<0.0001
Record keeping		
Yes: 66.7%, No; 33.3%	Yes: 51.7%, No: 48.3%	0.024
Routine administration of antimicrobials to newborns		
Yes: 24.5%, No: 75.5%	Yes: 10.0%, No: 90.0%	0.013
Means of calculating live bodyweight for the administration of pharmaceutical products		
Weighing: 20.6%, Estimation: 79.4%	Weighing: 33.3%, Estimation: 66.7%	0.027
Administration of flunixin in cases of clinical mastitis		
Yes: 9.9%, No: 90.1%	Yes: 1.7%, No: 98.3%	0.036
Quarantine of new animals arriving at the farm		
Yes: 63.8%, No: 36.2%	Yes: 45.0%, No: 55.0%	0.005
Presence of a fence or a wall around the farm		
Yes: 52.3%, No: 47.7%	Yes: 35.0%, No: 65.0%	0.012
Changes of rams/bucks into ewes/does during the mating period		
Yes: 28.6%, No: 71.4%	Yes:11.7%, No: 88.3%	0.005
Use of ultrasound for pregnancy diagnosis		
Yes: 33.9%, No: 66.1%	Yes: 15.0%, No: 85.0%	0.003
Grouping of pregnant females during the final stage of pregnancy		
Yes: 65.9%, No: 34.1%	Yes: 50.0%, No: 50.0%	0.017
Month of the start of the lambing/kidding season		
October	November	0.023
Administration of selenium to newborn animals		
Yes: 69.3%, No: 30.7%	Yes: 48.3%, No: 51.7%	0.001
Use of teat disinfection after milking		
Yes: 16.4%, No: 83.6%	Yes: 1.7%, No: 98.3%	0.003
Average quantity of hay provided daily to animals during the preceding season		
0.97 (1.18) kg	0.61 (1.00) kg	0.004
Provision of finished feed (concentrate) to adult animals throughout the year		
Yes: 31.9%, No: 68.1%	Yes: 53.4%, No: 46.6%	0.001
Type of finished feed (concentrate) provided to adult animals		
Pellets: 28.8%, Small pellets: 31.9%	Pellets: 51.7%, Small pellets: 15.5%	<0.01
Average quantity of finished feed (concentrate) provided daily to animals during the preceding season		
0.74 (0.62) kg	0.53 (0.48) kg	<0.0001
Person responsible for nutritional management		
Veterinarian: 30.5%	Veterinarian: 14.0%	0.003
Average milk production per ewe/doe during the preceding milking period		
199.5 (127.5) L	129.0 (114.0) L	<0.0001
Average number of lambs/kids born per ewe/doe		
1.28 (0.20) newborns	1.20 (0.16) newborns	0.003
Incidence of clinical mastitis during the preceding season		
2.0% (4.5%)	3.0% (4.1%)	0.021
Epg counts in faecal samples		
228 ± 11 epg	320 ± 42 epg	0.011
Body condition score		
2.40 (0.22)	2.26 (0.61)	0.16
Somatic cell counts		
0.543 × 10^6^ (0.504 × 10^6^–0.583 × 10^6^) cells mL^−1^	0.680 × 10^6^ (0.567 × 10^6^–0.814 × 10^6^) cells mL^−1^	0.026
Length of previous animal farming experience		
25 (25)	30 (10)	0.010
Daily period spent by farmer at the farm		
15 (7) h	10 (7) h	0.035
