# Supplementary material for: Evaluation of the Role of Veterinarians for Outcomes Related to the Health and Production of Dairy Small Ruminants in Greece
Source: Animals (Basel). 2023 Oct 30;13(21):3371. doi: 10.3390/ani13213371 (PMC10650737; doi:10.3390/ani13213371)
Supplement: Supplementary file 1 [file animals-13-03371-s001.zip › animals-2684115-supplementary.pdf]

Evaluation of the Role of Veterinarians for Outcomes Related to the Health and Production of Dairy Small Ruminants in Greece

Daphne T. Lianou and George C. Fthenakis

Table S1. Details of multivariable models employed for the evaluation of professional relationship with production- or health-related outcomes in 325 sheep flocks and 119 goat herds in Greece.

Outcome	Variables offered to the multivariable models (*n*)	Variables required in the final models
Professional relationship with a veterinarian	10	(a) Average annual milk production per ewe / doe, (b) Average number of lambs / kids born per ewe / doe, (c) Incidence of clinical mastitis, (d) Epg counts in faecal samples, (e) Body condition score, (f) Somatic cell counts in bulk-tank milk, (g) Isolation of staphylococci from bulk-tank milk
Average milk production per ewe / doe	3	(a) Age of the veterinarian
Somatic cell counts in bulk-tank milk	3	(a) Gender of the veterinarian, (b) Age of the veterinarian
Incidence of clinical mastitis	3	(a) Age of the veterinarian
Incidence of clinical mastitis	3	(a) Gender of the veterinarian, (b) Age of the veterinarian

Table S2. Results of univariable analysis of parameters (n = 106) evaluated for association, in 325 sheep flocks and 119 goat herds in Greece, with the outcome 'professional relationship with a veterinarian'.

**Farms in which there was a professional relationship with a veterinarian (n = 384)**				**Farms in which there was no professional relationship with a veterinarian (n = 60)**				
**General Management Applied in the Farm**								
Management system applied in farms								
Intensive	Semi-intensive	Semi-extensive	Extensive	Intensive	Semi-intensive	Semi-extensive	Extensive	*p*
48	155	157	24	5	14	20	21	< 0.0001
Seasonal transfer of animals to other site								
Yes		No		Yes		No		*p*
61		323		17		43		0.018
**Infrastructure**								
Availability of milking parlour								
Yes		No		Yes		No		*p*
292		92		29		31		< 0.0001
Availability of isolation facilities for animals								
Yes		No		Yes		No		*p*
296		88		33		27		0.0003
Availability of milk replacer facilities and equipment for administration of milk replacer								
Yes		No		Yes		No		*p*
20		364		3		57		0.95
**Animals**								
No. of female animals in the farm								
240 (100) ewes / does				250 (199) ewes / does				0.41
Average age of culling females								
≤ 6 years		> 6 years		≤ 6 years		> 6 years		*p*
239		145		30		30		0.007

**Health Management**										
Use of laboratory diagnostic examinations in samples of milk preventively										
Yes	No	Yes	No							*p*
91	293	4	56							0.0003
Use of laboratory diagnostic examinations in samples of blood preventively										
Yes	No	Yes	No							*p*
122	262	16	44							0.43
Use of laboratory diagnostic examinations in samples of faeces preventively										
Yes	No	Yes	No							*p*
51	333	0	60							0.003
Number of occasions of administration of anthelmintic drugs annually										
2 (1)	1.5 (1)									0.009
Families of anthelmintics administered										
Benzimidazoles	Imidazothiazoles	Macrocyclic lactones	Salicylanilides	Benzimidazoles	Imidazothiazoles	Macrocyclic lactones	Salicylanilides			*p*
287	9	281	31	51	1	37	4			0.08, 0.53, 0.07, 0.62
Pharmaceutical form of anthelmintics administered										
Tablet	Injectable solution	Oral drench	Pour-on solution	Premix	Tablet	Injectable solution	Oral drench	Pour-on solution	Premix	*p*
269	233	171	19	2	45	23	26	0	0	0.43, 0.001, 0.86, 0.08, 0.54
Administration of ectoparasiticides										
Yes	No	Yes	No							*p*
152	232	27	33							0.43
Vaccination against *Chlamydia* infection										
Yes	No	Yes	No							*p*
153	231	16	44							0.05

Vaccination against clostridial infections				
Yes	No	Yes	No	*p*
377	7	57	3	0.12
Vaccination against contagious agalactia				
Yes	No	Yes	No	*p*
235	149	16	44	< 0.0001
Vaccination against foot-rot				
Yes	No	Yes	No	*p*
4	380	1	59	0.67
Vaccination against contagious ecthyma				
Yes	No	Yes	No	*p*
4	380	0	60	0.43
Vaccination against paratuberculosis				
Yes	No	Yes	No	*p*
35	349	7	53	0.53
Vaccination against bacterial pneumonia				
Yes	No	Yes	No	*p*
162	222	21	39	0.29
Vaccination against staphylococcal mastitis				
Yes	No	Yes	No	*p*
150	234	10	50	0.0007
Total number of optional vaccines administered annually				
3 (2)		2 (2)		< 0.0001
Administration of 'dry-ewe' treatment at the end of the lactation period				
Yes	No	Yes	No	*p*
63	321	5	55	0.11

Duration of the dry-period				
3 (1.5) months		3 (2) months		0.46
Record keeping				
Yes	No	Yes	No	*p*
256	128	31	29	0.024
**Pharmaceutical Treatment**				
Routine administration of antimicrobials to newborns				
Yes	No	Yes	No	*p*
94	290	6	54	0.013
Maintenance of prescribed withdrawal periods after administration of pharmaceuticals				
Yes	No	Yes	No	*p*
378	6	60	0	0.33
Means of calculating live bodyweight for the administration of pharmaceutical products				
Weighing	Estimation	Weighing	Estimation	*p*
79	305	20	40	0.027
Routine overdosing				
Yes	No	Yes	No	*p*
71	313	16	44	0.14
Number of antibiotics used for treatment of clinical mastitis				
2 (0)		2 (0)		0.88
Route for administration of antibiotics				
Systemic	Intramammary	Systemic	Intramammary	*p*
271	32	43	6	> 0.50
Administration of flunixin in cases of clinical mastitis				
Yes	No	Yes	No	*p*
38	346	1	59	0.036

Biosecurity Management				
Quarantine of new animals arriving at the farm				
Yes	No	Yes	No	*p*
245	139	27	33	0.005
Isolation of sick animals at the farm				
Yes	No	Yes	No	*p*
328	56	46	14	0.08
Means for disposal of carcasses of animals that died in the farm				
Incineration / burial / removal by specialised agent	Given to dogs / left unburied / left in water streams	Incineration / burial / removal by specialised agent	Incineration / burial / removal by specialised agent	*p*
208	176	31	29	0.72
Presence of a ditch at the entrance of the farm				
Yes	No	Yes	No	*p*
0	384	0	60	n/r
Presence of a fence or a wall around the farm				
Yes	No	Yes	No	*p*
201	183	21	39	0.012
Carrying out disinfections in the farm				
Yes	No	Yes	No	*p*
350	34	50	10	0.06
Practicing sharing of equipment with other farms				
Yes	No	Yes	No	*p*
16	368	2	58	0.76
Administration of rodenticides				
Yes	No	Yes	No	*p*
149	235	28	32	0.25

Presence of spots suitable for reproduction of vectors within 500 m				
Yes	No	Yes	No	*p*
227	157	30	30	0.18
**Reproductive Management**				
Beginning of the mating period for ewes / does				
May		May		0.89
Application of reproductive control				
Yes	No	Yes	No	*p*
117	267	11	49	0.05
Changes of rams / bucks into the ewes / does during the mating period				
Yes	No	Yes	No	*p*
110	274	7	53	0.005
Use of artificial insemination				
Yes	No	Yes	No	*p*
0	384	0	384	n/r
Use of embryo transfer				
Yes	No	Yes	No	*p*
0	384	0	384	n/r
Use of ultrasound for pregnancy diagnosis				
Yes	No	Yes	No	*p*
130	254	9	51	0.003
Nutritional modifications before the lambing / kidding period				
Yes	No	Yes	No	*p*
260	124	37	23	0.36

Grouping of pregnant females during the final stage of pregnancy				
Yes	No	Yes	No	*p*
253	131	30	30	0.017
Administration of oxytetracycline to the pregnant animals				
Yes	No	Yes	No	*p*
193	191	23	37	0.09
Administration of selenium to pregnant animals				
Yes	No	Yes	No	*p*
122	262	12	48	0.06
Month of the start of the lambing / kidding season				
October		November		0.023
Induction of lambing / kidding				
Yes	No	Yes	No	*p*
4	380	2	58	0.15
Recording of births – Maintenance of a lambing / kidding book				
Yes	No	Yes	No	*p*
217	167	31	29	0.48
Newborn care and specific monitoring				
Yes	No	Yes	No	*p*
351	33	51	9	0.11
Administration of selenium to newborn animals				
Yes	No	Yes	No	*p*
266	118	29	31	0.001
Disinfection of navel stump in newborns				
Yes	No	Yes	No	*p*
245	139	32	28	0.12

Maintenance of a colostrum bank						
Yes	No		Yes	No		*p*
52	332		6	54		0.45
Tail docking in newborns						
Yes	No		Yes	No		*p*
245	139		33	27		0.19
Newborn fostering to female animals other than their dams						
Yes	No		Yes	No		*p*
270	114		37	23		0.18
Age of lamb / kid removal from their dams						
	50 (20) days			60 (15) days		0.12
**Management in the Milking Parlour**						
Daily number of milking sessions						
One	Two	Three	One	Two	Three	*p*
4	318	62	1	54	5	0.27
System pulsation rate						
< 140 p. min^{-1}	140 - 150 p. min^{-1}	> 150 p. min^{-1}	< 140 p. min^{-1}	140 - 150 p. min^{-1}	> 150 p. min^{-1}	*p*
69	168	55	9	16	4	0.61
System pressure						
< 38 kPa	38 - 42 kPa	> 42 kPa	< 38 kPa	38 - 42 kPa	> 42 kPa	*p*
25	234	33	2	24	3	0.94
Use of teat disinfection after milking						
Yes	No		Yes	No		*p*
63	321		1	59		0.003
Temperature of cleaning water after the milking sessions						
	70 (10) ^{o}C			70 (15) ^{o}C		0.81

Frequency of changing teatcups								
At least annually		Less frequently than annually		At least annually		Less frequently than annually		*p*
147		145		12		17		0.36
**Nutritional Management**								
Provision of hay as fodder to animals								
Yes		No		Yes		No		*p*
381		3		59		1		0.50
Average quantity of hay provided daily to animals during the preceding season								
0.97 (1.18) kg				0.61 (1.00) kg				0.004
Provision of straw to animals								
Yes		No		Yes		No		*p*
293		91		38		22		0.32
Provision of silage to adult animals								
Yes		No		Yes		No		*p*
83		301		7		53		0.07
Provision of finished feed (concentrate) to adult animals								
Yes		No		Yes		No		*p*
379		5		58		2		0.24
Provision of finished feed (concentrate) to adult animals throughout the year								
Yes		No		Yes		No		*p*
121		258		31		27		0.001
Type of finished feed (concentrate) provided to adult animals								
Mash	Pellets	Flakes	Small pellets	Mash	Pellets	Flakes	Small pellets	*p*
156	109	4	121	19	30	0	9	0.78, 0.0008, 0.43, 0.009
Average quantity of finished feed (concentrate) provided daily to animals during the preceding season								
0.74 (0.62) kg				0.53 (0.48) kg				< 0.0001

Person responsible for nutritional management								
Farmer themselves	Veterinarian	Animal nutritionist	Other professional	Farmer themselves	Veterinarian	Animal nutritionist	Other professional	*p*
247	117	15	77	45	7	0	11	0.11, 0.003, 0.11, 0.76
**Production- or Health-related Outcomes**								
Average milk production per ewe / doe during the preceding milking period								
199.5 (127.5) L				129.0 (114) L				< 0.0001
Average number of lambs / kids born per ewe / doe								
1.28 (0.20) newborns				1.20 (0.16) newborns				0.003
Incidence of clinical mastitis during the preceding season								
2.0% (4.5%)				3.0% (4.1%)				0.021
Incidence of abortion during the preceding season								
0.0% (3.1%)				0.0% (2.4%)				0.99
Incidence of lameness during the preceding season								
0.0% (2.0%)				0.0% (1.3%)				0.21
Incidence of mange during the preceding season								
0.0% (0.0%)				0.0% (0.0%)				0.60
Incidence of obstetrical problems during the preceding season								
0.0% (1.3%)				0.0% (1.2%)				0.71
Incidence of deaths, of any cause, in adult animals during the preceding season								
3.0% (3.9%)				3.0% (3.1%)				0.92
Incidence of pneumonia in lambs / kids during the preceding season								
0.0% (4.8%)				0.0% (5.4%)				0.45
Incidence of diarrhoea in lambs / kids during the preceding season								
2.0% (10.0%)				6.0% (16.7%)				0.08
Epg counts in faecal samples								
228 ± 11				320 ± 42				0.011

Body condition score				
2.40 (0.22)		2.26 (0.61)		0.016
Somatic cell counts in bulk-tank milk				
0.543×10^6 (0.504×10^6 - 0.583×10^6) cells mL^{-1}		0.680×10^6 (0.567×10^6 - 0.814×10^6) cells mL^{-1}		0.026
Total bacterial counts in bulk-tank milk				
428×10^3 (363×10^3 - 501×10^3) cfu mL^{-1}		561×10^3 (398×10^3 - 776×10^3) cfu mL^{-1}		0.23
Isolation of staphylococci from bulk-tank milk				
Yes	No	Yes	No	*p*
246	138	33	27	0.18
Isolation of antibiotic-resistant staphylococci from bulk-tank milk				
Yes	No	Yes	No	*p*
120	264	15	45	0.33
Isolation of *Listeria* spp. from bulk-tank milk				
Yes	No	Yes	No	*p*
4	380	0	60	0.42
Fat content in bulk-tank milk				
5.83% ± 0.06%		5.60% ± 0.16%		0.15
Protein content in bulk-tank milk				
4.12% ± 0.03%		4.02% ± 0.08%		0.24
Added water in bulk-tank milk				
0.57% ± 0.10%		1.01% ± 0.40%		0.14
**Characteristics of Human Resources**				
Age of the farmer				
46 (17)		50 (21)		0.53
Length of previous animal farming experience				
25 (25)		30 (10)		0.019

Farmer's general education						
Primary	Secondary or post-secondary	Tertiary	Primary	Secondary or post-secondary	Tertiary	*p*
24	266	46	6	48	6	0.58
Farmer's professional involvement in farming						
Full-time		Part-time	Full-time		Part-time	*p*
342		42	55		5	0.54
Daily period spent by farmer at the farm						
15 (7) hours			10 (7) hours			0.035
Family tradition in farming						
Yes		No	Yes		No	*p*
332		52	54		6	0.45
Presence of working staff in the farm						
Yes		No	Yes		No	*p*
138		246	19		41	0.52
Occurrence of brucellosis in farmer						
Yes		No	Yes		No	*p*
47		316	10		34	0.08

Table S3. Results of univariable analysis of parameters related to management practices and to production- or health-related outcomes (n = 39) evaluated for association, in 283 sheep flocks and 101 goat herds in Greece, with a 'professional relationship with a veterinarian', in accord with the gender of the veterinarian.

<table>
<tr><th colspan="5">Farms in which there was a professional relationship with a female veterinarian (n = 121)</th><th colspan="5">Farms in which there was a professional relationship with a male veterinarian (n = 263)</th><th></th></tr>
<tr><td colspan="11">Health Management</td></tr>
<tr><td colspan="11">Use of laboratory diagnostic examinations in samples of milk preventively</td></tr>
<tr><td colspan="3">Yes</td><td colspan="2">No</td><td colspan="3">Yes</td><td colspan="2">No</td><td>p</td></tr>
<tr><td colspan="3">38</td><td colspan="2">83</td><td colspan="3">53</td><td colspan="2">210</td><td>0.016</td></tr>
<tr><td colspan="11">Use of laboratory diagnostic examinations in samples of faeces preventively</td></tr>
<tr><td colspan="3">Yes</td><td colspan="2">No</td><td colspan="3">Yes</td><td colspan="2">No</td><td>p</td></tr>
<tr><td colspan="3">21</td><td colspan="2">100</td><td colspan="3">30</td><td colspan="2">233</td><td>0.11</td></tr>
<tr><td colspan="11">Number of occasions of administration of anthelmintic drugs annually</td></tr>
<tr><td colspan="5">2 (1)</td><td colspan="5">2 (1)</td><td>0.36</td></tr>
<tr><td colspan="11">Families of anthelmintics administered</td></tr>
<tr><td>Benzimidazoles</td><td>Imidazothiazoles</td><td>Macrocyclic lactones</td><td colspan="2">Salicylanilides</td><td>Benzimidazoles</td><td>Imidazothiazoles</td><td>Macrocyclic lactones</td><td colspan="2">Salicylanilides</td><td>p</td></tr>
<tr><td>89</td><td>1</td><td>100</td><td colspan="2">15</td><td>189</td><td>8</td><td>181</td><td colspan="2">16</td><td>0.78, 0.14, 0.005, 0.35</td></tr>
<tr><td colspan="11">Pharmaceutical form of anthelmintics administered</td></tr>
<tr><td>Tablet</td><td>Injectable solution</td><td>Oral drench</td><td>Pour-on solution</td><td>Premix</td><td>Tablet</td><td>Injectable solution</td><td>Oral drench</td><td>Pour-on solution</td><td>Premix</td><td>p</td></tr>
<tr><td>82</td><td>88</td><td>77</td><td>4</td><td>0</td><td>182</td><td>145</td><td>94</td><td>15</td><td>2</td><td>0.78, 0.001, < 0.0002, 0.13, 0.28</td></tr>
<tr><td colspan="11">Vaccination against Chlamydia infection</td></tr>
<tr><td colspan="3">Yes</td><td colspan="2">No</td><td colspan="3">Yes</td><td colspan="2">No</td><td>p</td></tr>
<tr><td colspan="3">46</td><td colspan="2">85</td><td colspan="3">107</td><td colspan="2">156</td><td>0.29</td></tr>
</table>

Vaccination against contagious agalactia				
Yes	No	Yes	No	*p*
74	47	161	101	0.99
Vaccination against staphylococcal mastitis				
Yes	No	Yes	No	*p*
49	72	101	162	0.70
Total number of optional vaccines administered annually				
3 (2)		3 (2)		0.99
Record keeping				
Yes	No	Yes	No	*p*
82	39	174	89	0.76
**Pharmaceutical Treatment**				
Routine administration of antimicrobials to newborns				
Yes	No	Yes	No	*p*
24	97	70	193	0.15
Means of calculating live bodyweight for the administration of pharmaceutical products				
Weighing	Estimation	Weighing	Estimation	*p*
31	90	46	215	0.07
Administration of flunixin in cases of clinical mastitis				
Yes	No	Yes	No	*p*
18	103	20	241	0.028
**Biosecurity Management**				
Quarantine of new animals arriving at the farm				
Yes	No	Yes	No	*p*
75	46	170	93	0.61

Isolation of sick animals at the farm				
Yes	No	Yes	No	*p*
102	19	226	37	0.67
Presence of a fence or a wall around the farm				
Yes	No	Yes	No	*p*
49	72	152	111	0.002
Carrying out disinfections in the farm				
Yes	No	Yes	No	*p*
118	3	232	31	0.003
**Reproductive Management**				
Application of reproductive control				
Yes	No	Yes	No	*p*
52	69	65	198	0.0003
Changes of rams / bucks into the ewes / does during the mating period				
Yes	No	Yes	No	*p*
35	86	75	188	0.93
Use of ultrasound for pregnancy diagnosis				
Yes	No	Yes	No	*p*
49	72	81	182	0.06
Grouping of pregnant females during the final stage of pregnancy				
Yes	No	Yes	No	*p*
95	36	158	105	0.015
Administration of oxytetracycline to the pregnant animals				
Yes	No	Yes	No	*p*
73	48	120	143	0.007

Administration of selenium to pregnant animals				
Yes	No	Yes	No	*p*
35	86	89	176	0.36
Month of the start of the lambing / kidding season				
October		October		0.11
Administration of selenium to newborn animals				
Yes	No	Yes	No	*p*
94	27	172	101	0.004
**Management in the Milking Parlour**				
Use of teat disinfection after milking				
Yes	No	Yes	No	*p*
19	102	44	219	0.80
**Nutritional Management**				
Average quantity of hay provided daily to animals during the preceding season				
0.83 (1.22) kg		1.04 (1.18) kg		0.59
Provision of silage to adult animals				
Yes	No	Yes	No	*p*
26	95	57	206	0.97
Provision of finished feed (concentrate) to adult animals throughout the year				
Yes	No	Yes	No	*p*
78	43	180	83	0.44

Type of finished feed (concentrate) provided to adult animals								
Mash	Pellets	Flakes	Small pellets	Mash	Pellets	Flakes	Small pellets	*p*
52	40	0	31	104	69	4	90	0.52, 0.17, 0.22, 0.09

Average quantity of finished feed (concentrate) provided daily to animals during the preceding season				
0.74 (0.54) kg		0.72 (0.62) kg		0.73

Person responsible for nutritional management								
Farmer themselves	Veterinarian	Animal nutritionist	Other professional	Farmer themselves	Veterinarian	Animal nutritionist	Other professional	*p*
76	40	6	26	171	77	9	51	0.67, 0.45, 0.47, 0.63
**Production- or Health-related Outcomes**								
Average milk production per ewe / doe during the preceding milking period								
190.0 (112.5) L				200.0 (131) L				0.43
Average number of lambs / kids born per ewe / doe								
1.25 (0.24) newborns				1.29 (0.22) newborns				0.40
Incidence of clinical mastitis during the preceding season								
1.7% (4.8%)				2.4% (4.6%)				0.16
Incidence of diarrhoea in lambs / kids during the preceding season								
1.4% (9.3%)				2.5% (10.1%)				0.44
Epg counts in faecal samples								
183 ± 15				250 ± 16				0.024
Body condition score								
2.36 (0.56)				2.42 (0.46)				0.55
Somatic cell counts in bulk-tank milk								
0.461×10^6 (0.401×10^6 - 0.529×10^6) cells mL^{-1}				0.585×10^6 (0.540×10^6 - 0.638×10^6) cells mL^{-1}				0.026

Table S4. Results of univariable analysis of parameters related to management practices and to production- or health-related outcomes (n = 39) evaluated for association, in 283 sheep flocks and 101 goat herds in Greece with a 'professional relationship with a veterinarian', in accord with the age of the veterinarian.

**Farms in which there was a professional relationship with a veterinarian (n = 384)**		
**Health Management**		
	r_{sp}	p
Use of laboratory diagnostic examinations in samples of milk preventively	–0.036	0.48
Use of laboratory diagnostic examinations in samples of faeces preventively	–0.063	0.34
Number of occasions of administration of anthelmintic drugs annually	0.071	0.17
Families of anthelmintics administered	0.010 < ǀvalueǀ < 0.091	> 0.07
Pharmaceutical form of anthelmintics administered	0.052 < ǀvalueǀ < 0.076	> 0.13
Vaccination against *Chlamydia* infection	0.006	0.91
Vaccination against contagious agalactia	0.111	0.029
Vaccination against staphylococcal mastitis	0.039	0.45
Total number of optional vaccines administered annually	–0.095	0.06
Record keeping	–0.044	0.39
**Pharmaceutical Treatment**		
	r_{sp}	p
Routine administration of antimicrobials to newborns	0.015	0.77
Means of calculating live bodyweight for the administration of pharmaceutical products	0.096	0.06
Administration of flunixin in cases of clinical mastitis	0.143	0.005
**Biosecurity Management**		
	r_{sp}	p
Quarantine of new animals arriving at the farm	–0.057	0.27
Isolation of sick animals at the farm	–0.090	0.08
Presence of a fence or a wall around the farm	–0.158	0.002

Carrying out disinfections in the farm	–0.072	0.16
**Reproductive Management**		
	r_{sp}	p
Application of reproductive control	0.074	0.15
Changes of rams / bucks into the ewes / does during the mating period	–0.022	0.66
Use of ultrasound for pregnancy diagnosis	0.054	0.29
Grouping of pregnant females during the final stage of pregnancy	–0.026	0.61
Administration of oxytetracycline to the pregnant animals	0.014	0.78
Administration of selenium to pregnant animals	0.185	0.0002
Month of the start of the lambing / kidding season	0.010	0.05
Administration of selenium to newborn animals	0.097	0.06
**Management in the Milking Parlour**		
	r_{sp}	p
Use of teat disinfection after milking	0.092	0.08
**Nutritional Management**		
	r_{sp}	p
Average quantity of hay provided daily to animals during the preceding season	–0.138	0.007
Provision of silage to adult animals	0.036	0.48
Provision of finished feed (concentrate) to adult animals throughout the year	0.012	0.82
Type of finished feed (concentrate) provided to adult animals	0.056	0.27
Average quantity of finished feed (concentrate) provided daily to animals during the preceding season	–0.115	0.025
Person responsible for nutritional management	0.039 < value < 0.074	> 0.15
**Production- or Health-related Outcomes**		
	r_{sp}	p
Average milk production per ewe / doe during the preceding milking period	–0.039	0.44
Average number of lambs / kids born per ewe / doe	0.022	0.66

Incidence of clinical mastitis during the preceding season	0.116	0.024
Incidence of diarrhoea in lambs / kids during the preceding season	0.013	0.79
Epg counts in faecal samples	0.131	0.020
Body condition score	0.070	0.17
Somatic cell counts in bulk-tank milk	–0.098	0.06

Table S5. Results of univariable analysis of parameters related to management practices and to production- or health-related outcomes (n = 39) evaluated for association, in 283 sheep flocks and 101 goat herds in Greece with a 'professional relationship with a veterinarian', in accord with the annual frequency of veterinary visits to the farm.

Farms in which there was a professional relationship with a veterinarian (n = 384)		
**Health Management**		
	r_{sp}	p
Use of laboratory diagnostic examinations in samples of milk preventively	–0.179	0.004
Use of laboratory diagnostic examinations in samples of faeces preventively	–0.238	< 0.0001
Number of occasions of administration of anthelmintic drugs annually	0.105	0.041
Families of anthelmintics administered	0.023 < value < 0.082	> 0.11
Pharmaceutical form of anthelmintics administered		
Tablet	–0.021	0.68
Injectable solution	–0.110	0.032
Oral drench	–0.096	0.06
Pour-on	–0.054	0.29
Premix	0.007	0.89
Vaccination against *Chlamydia* infection	–0.091	0.08
Vaccination against contagious agalactia	–0.143	0.005
Vaccination against staphylococcal mastitis	0.002	0.96
Total number of optional vaccines administered annually	0.025	0.63
Record keeping	0.066	0.20
**Pharmaceutical Treatment**		
	r_{sp}	p
Routine administration of antimicrobials to newborns	–0.105	0.040
Means of calculating live bodyweight for the administration of pharmaceutical products	–0.125	0.014
Administration of flunixin in cases of clinical mastitis	0.082	0.11

Biosecurity Management		
	r_{sp}	p
Quarantine of new animals arriving at the farm	–0.051	0.32
Isolation of sick animals at the farm	–0.042	0.41
Presence of a fence or a wall around the farm	–0.069	0.17
Carrying out disinfections in the farm	0.102	0.045
**Reproductive Management**		
	r_{sp}	p
Application of reproductive control	–0.083	0.10
Changes of rams / bucks into the ewes / does during the mating period	–0.161	0.002
Use of ultrasound for pregnancy diagnosis	–0.206	< 0.0001
Grouping of pregnant females during the final stage of pregnancy	0.076	0.14
Administration of oxytetracycline to the pregnant animals	0.025	0.63
Administration of selenium to pregnant animals	0.106	0.037
Month of the start of the lambing / kidding season	–0.078	0.13
Administration of selenium to newborn animals	–0.113	0.027
**Management in the Milking Parlour**		
	r_{sp}	p
Use of teat disinfection after milking	–0.058	0.25
**Nutritional Management**		
	r_{sp}	p
Average quantity of hay provided daily to animals during the preceding season	0.078	0.13
Provision of silage to adult animals	–0.252	< 0.0001
Provision of finished feed (concentrate) to adult animals throughout the year	–0.182	0.0004
Type of finished feed (concentrate) provided to adult animals	–0.111	0.030
Average quantity of finished feed (concentrate) provided daily to animals during the preceding season	–0.013	0.80

Person responsible for nutritional management		
Farmer themselves	0.210	0.0001
Veterinarian	–0.131	0.010
Animal nutritionist	–0.049	0.34
Other professional	0.011	0.82
**Production- or Health-related Outcomes**		
	r_{sp}	p
Average milk production per ewe / doe during the preceding milking period	–0.001	0.98
Average number of lambs / kids born per ewe / doe	0.091	0.08
Incidence of clinical mastitis during the preceding season	–0.069	0.18
Incidence of diarrhoea in lambs / kids during the preceding season	0.037	0.47
Epg counts in faecal samples	0.041	0.46
Body condition score	0.001	0.98
Somatic cell counts in bulk-tank milk	0.003	0.94
